# A Comparison of Pain between Parkinson's Disease and Multiple System Atrophy: A Clinical Cross-Sectional Survey

**DOI:** 10.1155/2019/3150306

**Published:** 2019-01-22

**Authors:** He-Yang You, Lei Wu, Hai-Ting Yang, Chen Yang, Xiao-Ling Ding

**Affiliations:** Department of Neurology, Provincial Hospital Affiliated to Anhui Medical University, Hefei, Anhui 230000, China

## Abstract

**Background:**

Pain is frequent in Parkinson's disease (PD) and Parkinson-plus syndrome. This study aimed to assess the prevalence, characteristics, therapy (especially the effect of dopaminergic therapy), and associated symptoms of pain in Parkinson's disease and multiple system atrophy (MSA) patients.

**Methods:**

Seventy-one PD patients, sixty-five MSA patients, and forty age-matched healthy controls were enrolled and evaluated by using the German pain questionnaire and visual analogue scale (VAS). In addition, the influence of pain in PD patients on anxiety, depression, and the quality of life was assessed with the Hospital Anxiety and Depression Scale (HADS) and Parkinson's Disease Questionnaire (PDQ-39).

**Results:**

Compared to that of the healthy controls, the PD and MSA patients had a significantly higher presence of pain (*P* < 0.01, *P* < 0.01). PD patients had a higher presence of pain than MSA patients (*P*=0.007). No difference in VAS scores was observed between the PD and MSA patients (*P*=0.148). A total of 21 PD patients (42.85%) with pain and 13 MSA patients (43.33%) with pain received treatment. A total of 13 PD patients with pain and 6 MSA patients with pain had an improved pain intensity after using dopaminergic medication. The differences in the disease duration, Hoehn and Yahr stages, and scores on the Unified Parkinson's Disease Rating Scale motor score, HAD-D, HAD-A, and PDQ-39 were significant between the PD patients with and without pain.

**Conclusion:**

PD and MSA patients are prone to pain with insufficient treatment. Pain interventions should be provided as soon as possible to improve the patient's life.

## 1. Introduction

Parkinson's disease (PD) is a common neurodegenerative disorder with a prevalence of approximately 1.7% in the population over 65 years of age in China. Pain is an important and common nonmotor symptom in PD, which has been frequently studied [1]. The prevalence rate of pain in PD varies from 40% to 85%, and there is an association between pain and depression and a decline in the quality of life [2]. Studies have found that pain is also frequent in Parkinson-plus syndrome, especially in patients with multiple system atrophy (MSA) [3]. However, comprehensive studies of the pain prevalence and specifics in MSA patients have rarely been performed.

A previous study showed that both PD and MSA patients were more likely to complain about pain than other Parkinson-plus disease patients. However, the differences in clinical characteristics and treatment of pain between PD and MSA patients have not been studied.

Quantitative measurement of pain is difficult because pain is a subjective symptom. In addition, the disparity of pain questionnaires contributes to the differences in the prevalence rate of pain in PD patients. Survey-based prevalence studies of PD have used short and simple questionnaires, such as the Brief Pain Inventory [4]. Therefore, after comparative studies of pain questionnaires, the German pain questionnaire (DSF) was selected to evaluate pain in this study. This questionnaire was used in a previous study to evaluate pain in PD patients [5].

Therefore, this study aimed to assess the prevalence, basic characteristics, and therapy (especially the effect of dopaminergic therapy) of pain in PD and MSA patients with the DSF and visual analogue scale (VAS). We also aimed to study the influence of pain on anxiety, depression, and quality of life with the Hospital Anxiety and Depression Scale (HADS) and Parkinson's Disease Questionnaire (PDQ-39) in PD patients with pain.

## 2. Materials and Methods

### 2.1. Patients

A total of 71 PD patients, 65 MSA patients, and 40 age-matched healthy controls (HC) were enrolled from the Anhui Provincial Hospital between January 2016 and September 2018. The PD diagnosis was based on the United Kingdom Brain Bank criteria [6]. The MSA diagnosis was based on the second consensus statement by the American Academy of Neurology [7]. For the MSA and PD patients, the exclusion criteria were as follows: cognitive impairment (MMSE < 26), treatment with neuroleptic drugs, patients suffering from another pain condition (rheumatic disease, traumatic, orthopaedic, or peripheral nerve injury), and patients with other causes of Parkinson syndrome, such as vascular Parkinson syndrome or medicine/toxin-induced Parkinson syndrome. Written informed consent was obtained from all subjects. The study was approved by the Ethics Committee of Provincial Hospital affiliated to Anhui Medical University.

### 2.2. Methods

The pain intensity was evaluated using the VAS. The VAS is designed to present to the respondents a rating scale with minimum constraints. Respondents mark the location on the 10-centimetre line corresponding to the amount of pain they have experienced.

The DSF is designed based on the principle of “Medical-Psychology-Society.” Compared with other more popular pain questionnaires, the DSF can be used to record the pain history more entirely and in a more orderly manner [8]. In this study, the DSF was used to record multidimensional experiences of pain, including pain sites, duration, intensity, pain-associated symptoms, pain relieving and intensifying factors, and pain improvement response to dopaminergic therapy [5].

Basic clinical characteristics were recorded, including age, gender, and disease duration. For the PD patients, disease severity was assessed according to Hoehn and Yahr (H&Y), and the motor disability and motor type were assessed using the Unified Parkinson's Disease Rating Scale motor score (UPDRS-III). We used the HADS to record depression and anxiety and the PDQ-39 to assess the quality of life in the PD patients. We divided the MSA patients into MSA-P and MSA-C groups.

## 3. Statistical Analysis

For descriptive analysis, quantitative parameters were expressed as mean ± standard deviation and qualitative parameters as frequency and percentage. Means were compared using *t*-test, and categorical data were compared using the chi-square test.

At first, pain presence and gender among the groups of PD patients, MSA patients, and healthy controls were analyzed using the chi-square test. Age and disease duration among the three groups were assessed by the independent-samples *t*-test.

Secondly, clinical and demographic characteristics between PD and MSA patients with pain were further compared. The statistical differences of VAS scores, age, and disease duration between the two groups were estimated using the independent-samples *t*-test. Gender differences between the two groups were compared by the chi-square test. We divided PD patients with pain into three types: tremor-dominant group, akinetic-rigid group, and equivalent group and divided MSA patients with pain into two types (MSA-P and MSA-C). The differences of pain presence of PD types and MSA types were analyzed with the chi-square test, respectively. And we analyzed the pain localization proportions and treatment in PD and MSA patients with pain by the means of bar chart.

At last, according to accompanied with or without pain, we divided PD patients into two groups and compared clinical and demographic characteristics. Age, disease duration, HY stage, the UPDRS-III, and scores of HAD-D, HAD-A, and PDQ-39 between the PD patients with and without pain were compared using independent-samples *t*-test.

SPSS version 22.0 was used to analyze the data. A two-tailed *P* value < 0.05 was considered significant.

## 4. Results

### 4.1. Study Population and Pain Presence

**Figure 1:**
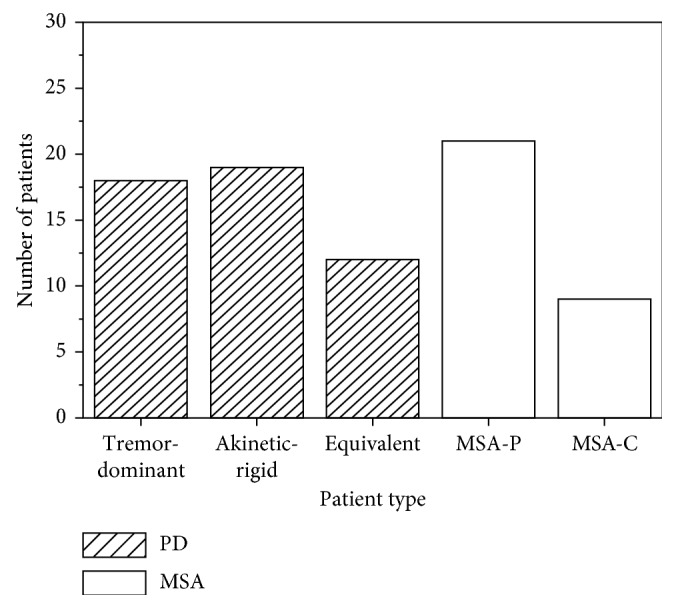
Number of patients with pain of different types in PD and MSA.

The demographic data and pain presence of the PD patients, MSA patients, and HC are presented in Table 1. Pain was reported in 49 PD patients (69.1%), 30 MSA patients (46.19%), and 6 healthy controls (15%). Compared to that of the HC, the PD and MSA patients had a significantly higher pain presence (*P* < 0.01, *P* < 0.01). And PD patients had a higher presence of pain than MSA patients (*P*=0.007). According to clinical features, we divided 49 PD patients with pain into three types (18 tremor-dominant, 19 akinetic-rigid, and 12 equivalent) and 30 MSA patients with pain into two types (21 MSA-P and 9 MSA-C). No difference was observed in the presence of pain among the PD types. And no difference was observed in the presence of pain between the MSA-P and MSA-C groups (*X*^2^=1.087, *P*=0.297).  Figure 1 shows the number of patients with pain of different types in PD and MSA.

### 4.2. Pain Intensity, Therapy, and Localization between PD and MSA Patients with Pain

The means of VAS scores of PD and MSA patients with pain were 5.08 ± 1.98 and 4.47 ± 1.60, respectively. There was no difference of VAS scores between the two groups (*P*=0.148). Compared to that of the MSA patients with pain, the PD patients with pain had longer disease durations (*P*=0.008).  Table 2 presents more details about the PD and MSA patients with pain.

**Figure 2:**
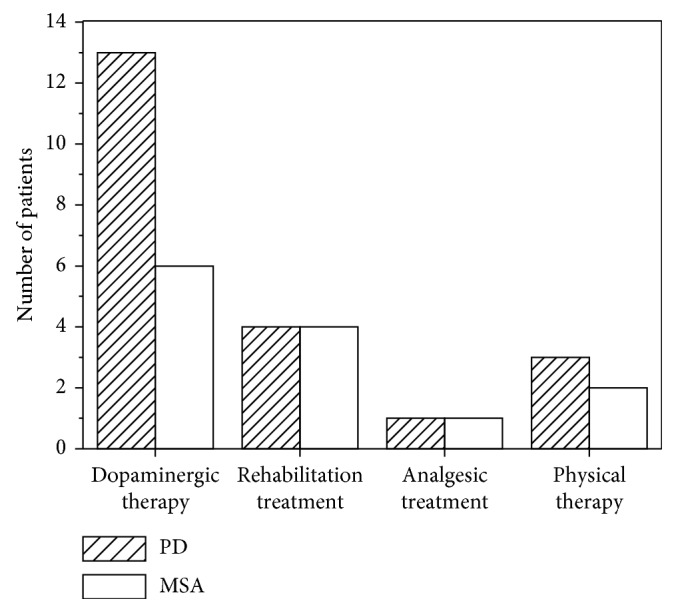
Treatments in PD and MSA patients with pain.

Figure 2 presents treatments of PD and MSA patients with pain with bar chart. A total of 21 PD patients with pain (42.86%) received therapy, of whom 13 patients improved their pain symptoms with dopaminergic therapy. The other PD patients with pain received rehabilitation treatment, physical therapy, and analgesic treatment (OTC medicine). A total of 13 MSA patients with pain (43.33%) received therapy, 5 patients with levodopa treatment, 1 patient with pramipexole treatment, 4 patients with rehabilitation treatment, 1 patient with analgesic treatment (OTC medicine), and 2 patients with physical therapy. No patients with pain were treated with antidepressant.

**Figure 3:**
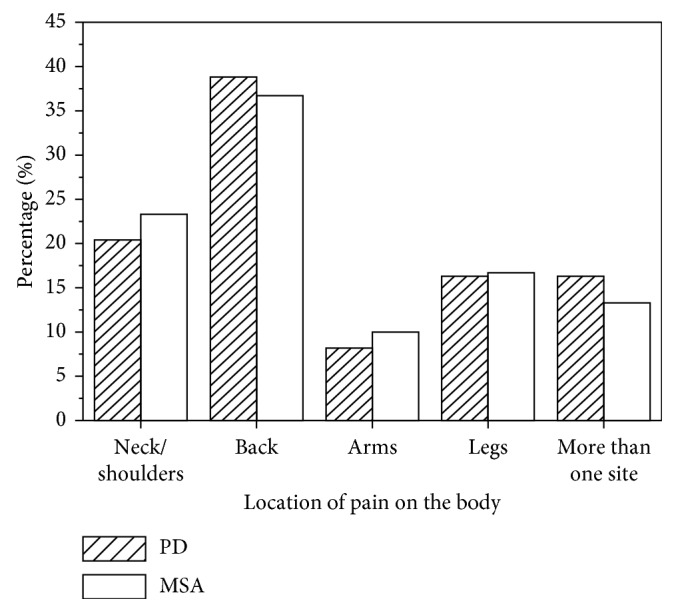
Localization proportions in PD and MSA patients with pain.

The most common pain location in the PD patients was back pain (38.8%), followed by neck or shoulder, multiple sites, legs, and arms pain. The top two most common pain locations in the MSA patients were back pain (36.7%) and neck or shoulder pain (23.3%).  Figure 3 presents pain localization of the PD and MSA patients with pain with bar chart.

### 4.3. Clinical Characteristics of the PD Patients with and without Pain

Clinical characteristics of the PD patients with and without pain were analyzed. PD patients with pain had longer disease duration than PD patients without pain (4.37 ± 3.01 and 3.29 ± 4.40, respectively, *P*=0.018). And there were differences of HY stages and UPDRS-III scores between the two types (*P* < 0.01, *P*=0.002). In addition, differences of scores on the HAD-A, HAD-D, and PDQ-39 were significantly different between the PD patients with and without pain (*P*=0.009, *P*=0.003, *P*=0.001, respectively) (Table 3).

## 5. Discussion

Our study found that pain was prevalent in PD and MSA patients. However, only approximately half of the PD or MSA patients with pain received treatment, suggesting that the current treatment of pain associated with Parkinson's syndrome was not valued and that no effective and specific treatment was available. PD patients with pain are more likely to be anxious and depressed and have a reduced quality of life than PD patients without pain.

### 5.1. Pain and Prevalence

We found that 69.1% of the PD patients suffered from pain. At present, the pathological mechanism of pain is still unclear. The basal ganglia integrate incoming nociceptive information and contribute to coordinated motor responses in pain avoidance and nocifensive behaviors. Nigral and extranigral pathology involving the cortical areas, brainstem nuclei, and spinal cord may contribute to abnormal central nociceptive processing in PD patients with or without pain [9]. A functional remodulation of pain processing pathways occurs in the absence of clinically overt pain symptoms in drug‐naive PD patients. These mechanisms may eventually become dysfunctional over time, contributing to the emergence of pain symptoms in the more advanced PD stages [10]. A meta-analysis that reviewed 22 studies with 616 PD patients and 451 HC supported the finding that PD patients had increased pain sensitivity, which might be due to dopaminergic and regional mechanisms [11].

We found that 30 MSA patients (46.15%) suffered from pain. There was a trend towards a higher prevalence in MSA-P compared to MSA-C patients although the difference was not significant, which might be due to the small sample size. Few studies have investigated the pain mechanism in MSA patients. Several brain regions are neurodegenerative in MSA, such as the thalamus and the locus coeruleus, resulting in striatal neurodegeneration and changes in dopaminergic system function. Thus, pain in MSA results from striatal neurodegeneration. MSA patients presented alteration of pain perception with both lower subjective and objective pain thresholds than those of healthy controls [12]. In addition, Perrotta et al. [13] reported that MSA patients showed a significant reduction in the temporal summation threshold of the nociceptive withdrawal reflex compared to that of healthy subjects.

In our study, there was difference in the pain presence between the PD and MSA patients with pain. PD patients had a higher presence of pain than MSA patients (*P*=0.007). The result was inconsistent to that of a recent study with 65 PD patients and 21 MSA patients [14]. However, this result should be further verified with data from a larger sample. Another recent study comprising 28 patients (14 PD and 14 MSA-P) found that 78.6% of the multiple system atrophy patients and 37.5% of the Parkinson's disease patients experienced pain. An imaging study reported that the lateral nigra was targeted in both MSA-P and PD. However, a pathological series suggested greater involvement of the medial nigra in MSA-P based on the lower levels of dopamine terminal function in the anterior putamen and head of the caudate in MSA-P relative to PD [15]. Moreover, pathological lesions and metabolic dysfunction affecting the brainstem that were observed in both diseases were more severe in the MSA than in the PD patients based on [123I]*β*-CIT SPECT images, with greater atrophy of the middle cerebellar peduncle, the cerebellum or the pons (including the locus coeruleus), and hypometabolism in the brainstem [16].

### 5.2. Pain and Treatment

In this study, 21 PD patients (42.85%) with pain and 13 MSA patients (43.33%) with pain received treatment. A total of 13 PD patients with pain were diagnosed with PD relevant pain, whereas the others improved their pain using massage or pain killers. The low rate of clinical treatment for pain may be related to the lack of effective treatment for pain. For PD patients with pain, there are two pain treatment methods (drug and nondrug treatment). For pharmacological therapy, pain can be significantly alleviated or abolished by adjustment of dopaminergic medication [17]. In our study, we found one case that achieved pain reduction with the use of pramipexole. Patients treated with pramipexole reported a reduction of pain during the “on-period” [18]. However, in a double-blind, placebo-controlled trial, Barone et al. [19] found no significant difference in the effects on pain between pramipexole and a placebo. Additionally, rotigotine was able to achieve a numerical improvement in pain intensity in patients with advanced-stage PD [20]. Other medicines, such as subcutaneous apomorphine [21] or prolonged-release oxycodone-naloxone [22], need to be validated by future clinical studies. For nondrug treatment, the most efficient method to improve pain intensity is deep brain stimulation (DBS). All pain scores were significantly improved 12 months after STN-DBS, which was not correlated with motor improvement, depression scores, or L-Dopa reduction [23]. Additionally, some PD patients have pain due to limb stiffness, and thus regular exercise may be useful for improvement of pain.

For MSA patients, few studies have found useful treatment. In our study, we found that 6 MSA patients with pain improved their pain intensity through the use of dopaminergic medication, including levodopa and pramipexole. Therefore, although dopaminergic medication is not useful for motor symptoms, it can be experimentally used for the treatment of pain for MSA patients with pain. Four MSA patients with pain in our study improved their pain intensity with regular rehabilitation exercise, which showed that regular exercise might be another pain treatment method.

### 5.3. Pain and Location

In this study, the predominant sites of pain in the PD and MSA patients were the back and the neck and shoulder, which were considered to be skeletal muscle pain by Ford [24]. Skeletal muscle pain is caused by stiffness of the limbs resulting in reduced joint or limb movement, an abnormal posture gait and joint muscle traction. Joint muscle traction causes non-nervous tissue inflammatory damage, resulting in joint pain and muscle soreness and tightness. “Icy shoulder” may be the first symptom of PD. However, our study suggests that pain in the back or paraspinal muscles should not be neglected in clinical practice and may also be a prodrome to PD.

Pain is a nonmotor symptom that is somewhat neglected. In this study, we found that anxiety and depression were more likely to occur when PD patients suffered from pain and that the quality of life declined. Therefore, formulating an effective treatment for pain in PD and MSA can largely improve a patient's emotional state and quality of life.

Although the DSF scale is not a widely accepted questionnaire for PD patients with pain globally, our study selected the DSF scale as a tool to evaluate features of pain. The DSF is an effective and reliable tool that records the multidimensional experiences of patients with pain. The pain of more than 85% of patients can be fully assessed using this questionnaire. A high degree of agreement was found between clinical and psychosocial diagnoses of pain and the patients' pain data collected by the DSF. Therefore, doctors can adjust the treatment of pain according to the patient's pain clinical characteristics, basic psychological state, and influence on social work collected by the DSF [5, 8].

This study has some limitations. First, this study is a cross-sectional observational and retrospective study. Second, the selected population and the number of patients have limitations. In addition, the pain score of the study was evaluated using questionnaires, which can easily be affected by subjective factors. Our follow-up plan is to select quantitatively detectable pain thresholds and obtain more objective results.

## 6. Conclusions

This study found that PD and MSA patients often experienced pain. However, treatment of pain associated with Parkinson's syndrome has not been taken seriously. Clinicians should treat pain as seriously as the motor symptoms of Parkinson's syndrome and take corresponding measures for the treatment of pain to improve the patients' quality of life.

## Figures and Tables

**Table 1:** Demographic data and presence of pain for the PD patients, MSA patients, and healthy controls.

	PD	MSA	HC	PD vs MSA (*P* value)	PD vs HC (*P* value)	MSA vs HC (*P* value)
Number of subjects (sex ratio)	71 (39 men, 32 women)	65 (43 men, 22 women)	40 (24 men, 16 women)	0.181^a^	0.691^a^	0.538^a^
Age in years	59.72 ± 9.38	62.65 ± 8.13	62.83 ± 7.17	0.055^b^	0.072^b^	0.909^b^
Disease duration in years	4.03 ± 3.50	2.35 ± 2.10		<0.01^b^^*∗*^		
Pain frequency, *n* (%)	49 (69.1%)	30 (46.19%)	6 (15%)	0.007^a^^*∗*^	<0.01^a^^*∗*^	<0.01^a^^*∗*^

^a^Chi-square test. ^b^Independent-samples *t*-test. ^*∗*^*P* < 0.05.

**Table 2:** Pain intensity and therapy in PD and MSA patients with pain.

	PD patients with pain	MSA patients with pain	Test value	*P* value
Number of subjects (sex ratio)	49 (26 men, 23 women)	30 (19 men, 11 women)	0.437^a^	0.509^a^
Age in years	60.10 ± 8.48	63.3 ± 8.40	−1.65^b^	0.103^b^
Disease duration in years	4.37 ± 3.01	2.56 ± 2.67	2.707^b^	0.008^b^^*∗*^
VAS (1–10)	5.08 ± 1.98	4.47 ± 1.60	1.46^b^	0.148^b^
Therapy for pain	21	13		
Pain improvement with dopaminergic therapy	13	6		

VAS, visual analogue scale. ^a^Chi-square test. ^b^Independent-samples *t*-test. ^*∗*^*P* < 0.05.

**Table 3:** Clinical characteristics of the PD patients with and without pain.

	PD patients with pain	PD patients without pain	Test value	*P* value
Number of subjects (sex ratio)	49 (26 men, 23 women)	22 (13 men, 9 women)	0.223^a^	0.637^a^
Age in years	60.10 ± 8.48	58.86 ± 11.31	0.512^b^	0.61^b^
Disease duration in years	4.37 ± 3.01	3.29 ± 4.40	2.359^b^	0.018^b^^*∗*^
HY stage	2.64 ± 0.89	1.68 ± 0.50	4.312^b^	<0.01^b^^*∗*^
UPDRS-III	26.33 ± 11.77	17.95 ± 11.82	3.037^b^	0.002^b^^*∗*^
HAD-A	6.20 ± 2.97	4.47 ± 2.35	2.596^b^	0.009^b^^*∗*^
HAD-D	5.84 ± 2.75	3.95 ± 2.59	2.976^b^	0.003^b^^*∗*^
PDQ-39	43.37 ± 23.05	24.68 ± 11.19	3.600^b^	0.001^b^^*∗*^

HY stage: Hoehn and Yahr stage; UPDRS-III: Unified Parkinson's Disease Rating Scale motor score; HAD-D: the depression scores of the Hospital Anxiety and Depression Scale; HAD-A: the anxiety scores of the Hospital Anxiety and Depression Scale; PDQ-39: the Parkinson's Disease Questionnaire. ^a^Chi-square test. ^b^Independent-samples *t*-test. ^*∗*^*P* < 0.05.

## Data Availability

The data used to support the findings of this study are available from the corresponding author upon request.

## Abbreviations

PD: Parkinson's disease
MSA: Multiple system atrophy
DSF: German pain questionnaire
VAS: Visual analogue scale
HADS: Hospital Anxiety and Depression Scale
PDQ-39: Parkinson's Disease Questionnaire
HC: Healthy controls
H&Y: Hoehn and Yahr stage
UPDRS-III: Unified Parkinson's Disease Rating Scale motor score
DBS: Deep brain stimulation.
